# Characterization of chemical and carbon isotopic compositions of gases during thermochemical sulfate reduction and implications for gas origin and content

**DOI:** 10.1038/s41598-022-13017-3

**Published:** 2022-06-01

**Authors:** Huijuan Guo, Min Liu, Yunpeng Wang, Qiang Wang, Jinzhong Liu, Ping’an Peng

**Affiliations:** 1 State Key Laboratory of Organic Geochemistry, Guangzhou Institute of Geochemistry, Chinese Academy of Sciences, Guangzhou, 510640 China; 2 CAS Center for Excellence in Deep Earth Science, Guangzhou, 510640 China; 3 University of Chinese Academy of Sciences, Beijing, 100049 China

**Keywords:** Solid Earth sciences, Geochemistry

## Abstract

For identifying the occurrence and extent of thermochemical sulfate reduction (TSR) reaction of natural gas and better understanding the chemical and carbon isotopic variations in natural gas reservoirs, high-pressure hydro-pyrolysis with a special designed apparatus was performed using natural gas and various amounts of MgSO_4_·7H_2_O at up to 360 °C. The yields, chemical and isotopic compositions of the gases produced during TSR and thermal cracking were measured. As the extent of TSR reaction increased, the concentrations of CH_4_, CO_2_ and H_2_S increased in a nonlinear way, while those of C_2_H_6_ and C_3_H_8_ decreased. According to the variation of gas content, the TSR reaction of alkane gases can be divided into an uncatalyzed and a catalyzed stage, which is different from previous studies that treated the TSR reaction of alkane gases as a non-autocatalytic reduction process. As the concentration of MgSO_4_·7H_2_O increased, the rate of TSR reaction with hydrocarbon gases increased. The concentrations of HSO_4_^−^ and volume of aqueous phase could be responsible for the different TSR reaction rates in the catalyzed stage. The co-variation of ln(C_1_/C_2_) and ln(C_2_/C_3_) could be related to the TSR reaction of alkane gases. Our study provides clues for understanding the compositional variations in natural conditions.

## Introduction

H_2_S gas is corrosive to petroleum processing equipment, harmful to the environment, dangerous to humans and affects the quality of hydrocarbon^1,2^. Thermochemical sulfate reduction (TSR) is considered one of the most important processes for producing abundant H_2_S in carbonate reservoirs^3^. TSR is a redox reaction between sulfates and organic matter to generate CO_2_, H_2_S, organic sulfur compounds, diamondoids and solid bitumen^4–8^. Geological observations have suggested various temperature thresholds for the occurrence of TSR, ranging from a minimum of 80 ℃ to a much higher value of 180 ℃^5,9^. The great discrepancy in the onset temperature for TSR can be attributed to regional variations in its controlling factors^5,9^. Many laboratory studies have been conducted on the mechanism of TSR and the factors that could influence its reaction rate^10–13^. Through conducting TSR reactions involving C_21_ to C_35_ normal alkanes, a two-stage reaction mechanism has been proposed^11^. The initial sulfate reduction reaction is slow and non-autocatalytic until a threshold concentration of H_2_S is reached, at which point the catalyzed sulfate reduction reaction becomes dominant. Kinetic parameters were determined for sulfate reduction by hydrocarbons (C_21_ to C_35_) without the initial presence of low-valent sulfur. The reaction rate in the uncatalyzed stage, which relates to the direct oxidation of hydrocarbons by aqueous sulfate, is significantly slower than that in the catalyzed stage. Therefore, the uncatalyzed stage can be considered as the rate-determining step of TSR. As for the reaction in the uncatalyzed stage of TSR, previous studies have mainly dealt with liquid hydrocarbons. The types of functional groups present in liquid hydrocarbons can significantly affect the extent of the TSR reaction^14^. Experimental results have shown that the relative reactivity during TSR is 1-octene > 1-octanol > 1-octanone > *n*-octane > octanoic acid > octylbenzene > xylene. For saturated hydrocarbons, the relative reactivity during TSR is long chain iso-alkanes > n-alkane > cycloalkanes > C_2_–C_4_ alkanes > methane^15^. Besides the hydrocarbons involved in the TSR reaction, aqueous chemistry can also affect the oxidation rate of hydrocarbons by sulfate^9,10,16,17^. Zhang et al.^10^ investigated the effect of pH adjusted by mineral buffers on TSR with H_2_S as the initiator. Some other authors have studied the effects of the concentrations of HSO_4_^−^, contact ion pairs (CIPs), and salts on the initiation of TSR involving *n*C_16_ hydrocarbons. Their results showed that increasing in the concentrations of all three substances can accelerate the TSR reaction rate^9,16^.

Natural TSR is generally observed in petroleum accumulations that contain crude oil and condensate^5^. There are also cases of TSR involving wet gas and dry gas^1,3,5,7,18–21^. Moreover, the TSR reaction involving hydrocarbon gases might also occur in shale gas reservoirs^22,23^. In these gas reservoirs, labile sulfur compounds (LSC) might not be present. For gas reservoirs without the initial presence of low-valent sulfur, it is necessary to determine the kinetic parameters of sulfate reduction by hydrocarbon gases and the influence of the TSR reaction of these gases on the chemical and isotopic compositions of gas mixtures in order to estimate the occurrence and extent of TSR reaction of gases. In a previous study, TSR reactions of gaseous hydrocarbons were carried out and kinetic parameters were calculated^24^. However, this study was carried out at temperatures higher than the supercritical conditions for water, at which the property of water can change greatly, and it may not exist in the liquid phase where the TSR reaction of alkane gases occurs. In addition, the variation in the carbon isotopic composition of gaseous products was not investigated. Based on the laboratory data reported by Pan et al.^25^, Xia et al.^26^ proposed that TSR by gaseous hydrocarbons is a kinetically-controlled non-autocatalytic process and that the lack of apparent autocatalysis is due to the absence of the required intermediate species or their non-reactivity with active sulfate. In Pan’s work, high molecular weight aromatic compounds and H_2_S were present in the initial gaseous mixture used to carry out the TSR reaction, as it was generated from kerogen containing 3.5% S. H_2_S can significantly catalyze sulfate reduction and enhance the rate of TSR by forming LSC through its reaction with oils or gaseous hydrocarbons. The onset temperature of TSR decreased significantly and the reaction rate was accelerated with the increase of LSC content^10,27^. Pyrolysis reaction without LSC among the initial reactants could have a very different result.

Chemical and carbon isotopic compositions of gaseous hydrocarbons have been used as key indicators to differentiate the origins of gaseous hydrocarbons, their mode of generation, thermal maturity levels, and filling history of reservoirs^25,28^. However, these parameters of gaseous hydrocarbons can also be influenced by other processes, such as TSR, as proven by laboratory experiments and geological observations^1,19,29–31^. The influences of TSR on many geochemical parameters of natural gas, such as C_1_/(C_*n*_H_2*n*+2_), ln(C_1_/C_2_), and δ^13^C of methane and ethane, are similar to those of thermal maturation^29,30^. Previous studies have used the gas souring index H_2_S/(H_2_S + ΣC_*n*_H_2*n*+2_) and the δ^13^C of alkane gases as indicators of the extent of TSR^1,19,31^. H_2_S could react with the surrounding calcite or dolomite and precipitate as pyrite, while the δ^13^C value of alkane gases would become less negative as the extent of TSR increased. To constrain the occurrence and extent of the TSR reaction, some other criteria should be used. The ln(C_1_/C_2_) versus ln(C_2_/C_3_) could be used to distinguish hydrocarbon gases generated through the primary cracking of kerogen from gases generated by the secondary cracking of oils^28^. The value of ln(C_2_/C_3_) is almost constant during the primary cracking of kerogen, whereas it increases drastically during the secondary cracking of oils. In contrast, the value of ln(C_1_/C_2_) increases progressively during the primary cracking and is constant during secondary cracking^28^. In the sour natural gas reservoirs from northeastern Sichuan Basin, the value of ln(C_2_/C_3_) first increased and then decreased with increasing ln(C_1_/C_2_)^1^. It was explained that the decrease of ln(C_2_/C_3_) might be related to the mixing of primary cracking gas^1^. In the TSR reaction involving mainly wet gas and small amount of condensate and kerogen (prepared by the pyrolysis of kerogen under 450 ℃ for 72 h) conducted by Pan et al.^25^, the value of ln(C_2_/C_3_) first increased and then decreased with increasing ln(C_1_/C_2_). This indicated that the TSR reaction of wet gas and/or small amount of condensate and highly-mature kerogen were responsible for the decrease of ln(C_2_/C_3_). For gas reservoirs with wet gas and no condensate and kerogen, whether the ln(C_2_/C_3_) would decrease is still a question. This question could be answered by performing pyrolysis simulations of TSR reaction involving wet gas in the laboratory.

For natural gas produced from Lower Triassic and Permian gas reservoirs in the northwestern Sichuan Basin in China, an unusual partial reversal (δ^13^C_1_ > δ^13^C_2_) of the normal relationship of increasing δ^13^C with increasing alkane chain length occurs when the gas souring index H_2_S/(H_2_S + ΣC_*n*_H_2*n*+2_) is less than 0.01. When the gas souring index is higher than 0.01, the δ^13^C partial reversal is canceled, showing that ethane is more reactive than methane during TSR^1,30^. Reversed carbon isotope values are usually explained by the the mixing of the gas generated from different sources and/or at different maturity levels^32,33^. Liu et al.^34^ performed the TSR reaction of *n*-C_9_ hydrocarbons and showed that partial δ^13^C value reversal occurred initially, but with increasing TSR extent, the value of δ^13^C_2_ increased and the partially reversed isotopic sequence changed to a positive sequence. This may indicate that the partial δ^13^C value reversal occurs in the uncatalyzed stage of the TSR reaction. However, as far as we know, the δ^13^C value variation of alkane gases produced in the initial stage of the TSR reaction involving natural gas has not hitherto been investigated.

In this study, hydro-pyrolysis involving natural gas and MgSO_4_ was performed to investigate variations in the chemical and isotopic compositions of the gas products during the TSR reaction. This could provide clues for identifying the occurrence and extent of TSR reaction of natural gas and contribute to a better understanding of the chemical and carbon isotope variation in gas reservoirs. Accordingly, this work could help to reduce the risks associated with the exploration of sour natural gas reservoirs.

## Experiment and methods

### Quantitative loading of hydrocarbon gas into a gold tube reactor

**Figure 1:**
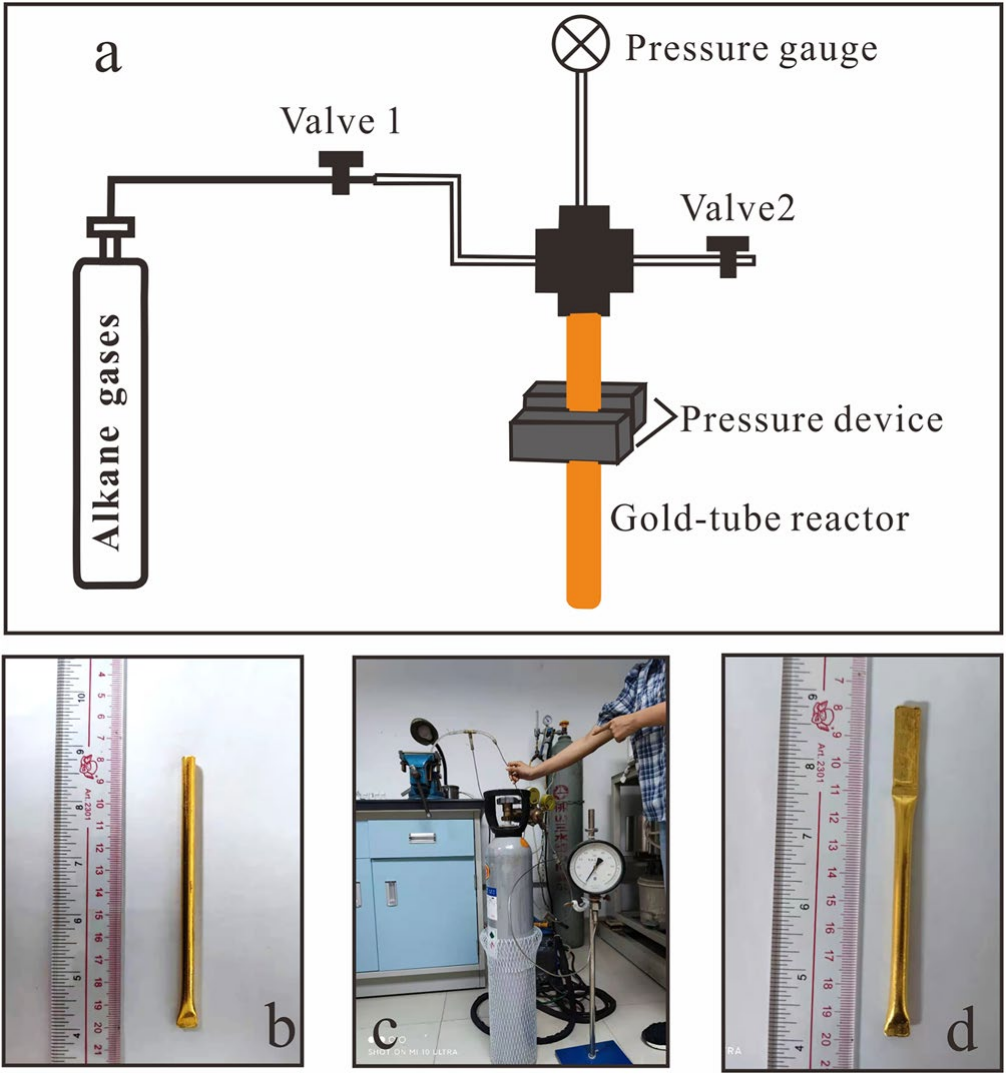
Schematic diagram of loading alkane gases into gold-tube reactors (**a**), picture of the gold tube with one end welded (**b**), picture of the device used to load gas into the gold tube (**c**), and picture of the gold tube after loading gas into and being welded in the other end (**d**).

Gold tubes served as inert vessels for TSR pyrolysis. Each gold tube was about 100 mm long, with an internal diameter of 5.4 mm and a wall thickness of 0.3 mm. One end of each tube was firstly crimped with an argon arc-welder (Fig. 1b). The tubes were then heated to 800 °C to remove any residual organic material. Then, MgSO_4_·7H_2_O (600 mg) was accurately weighed and transferred to each tube through a small glass funnel with an external diameter slightly smaller than the internal diameter of the tubes. Deionized water (100 μL) was then pipetted into each tube. Although anhydrite might be the reactive oxidant in natural TSR reservoirs, it is usually not used in laboratory TSR studies due to its low solubility. In natural situations, magnesium is always present and may catalyze TSR reaction^13^. When temperatures exceeded 200 °C, solutions containing Mg^2+^ and SO_4_^2−^ form a magnesium–hydroxide–sulfate–hydrate complex. As a result, the dominant aqueous sulfate species shift from MgSO_4_(aq) to HSO_4_^−^^35^. Theoretical studies showed that the activation energy for the reduction of HSO_4_^−^ (54.21 kcal /mol) by hydrocarbons is comparable with that for the reduction of MgSO_4_ contact ion-pair ([MgSO_4_]_CIP_) (54.95 kcal/mol) by hydrocarbons^13^. It is possible that high-temperature studies of TSR could be conducted on aqueous solutions containing HSO_4_^−^ to derive the rate constant for TSR under various conditions.

A special apparatus was developed to load gaseous reactants into gold tubes, as shown in Fig. 1a,c. Each tube was first flushed five times with the gaseous reagents to remove the air. The gaseous reactants were then introduced into the tubes at 0.5–0.8 MPa and kept at equilibrium for 5 min. Each tube was clamped at a point approximately 80–90 mm above the bottom and held by a compression device (Fig. 1c,d). It was then cut just below the nut connecting it to the gas channel, and its open end was welded with an argon arc welder while the other end was immersed in a liquid nitrogen trap. In this way, four gold tubes were prepared with equal amounts of solid and liquid reactants. Without heating the tubes, the gas was recovered and analyzed according to the procedure described in “Chemical and isotopic analysis” section. The amount of gases introduced into the gold tube was calculated based on the gas pressure during loading and the effective volume of the gold tube, assuming ideal gas behavior (PV = nRT). Comparative results showed that the recovered amounts of gaseous reactants were within about 4% of the amount introduced (Table 1), indicating that the method for introducing gaseous reactants into gold tubes is accurate and practical.

**Table 1:** The compositions and/or carbon isotope ratios of gaseous hydrocarbons collected from four small gold tubes.

Sample name	Measured gas amount	Calculated gas amount	Relative error	Gas volume percent				δ^13^C		
				CH_4_	C_2_H_6_	C_3_H_8_	N_2_	CH_4_	C_2_H_6_	C_3_H_8_
	(μmol)	(μmol)	(%)	%	%	%	%	‰	‰	‰
Gas-E-1	641.96	656.70	− 2.34	25.89	30.10	24.64	19.38	nm	nm	nm
Gas-E-2	591.52	602.23	− 1.82	25.22	29.80	24.73	20.25	nm	nm	nm
Gas-E-3	654.02	662.95	− 1.35	25.66	30.34	25.11	18.89	− 33.28	− 26.63	− 28.34
Gas-E-4	675.45	662.95	1.87	25.19	29.79	24.99	20.04	− 33.31	− 26.78	− 28.34

*nm* not measured.

### Hydrous pyrolysis

After the gold tubes were loaded with the reagents, they were placed in separate stainless-steel autoclaves, which in turn were placed in a pyrolysis oven. The pyrolysis experiments were performed under isothermal conditions. Each autoclave was heated to 360 °C (below the critical temperature of water of 374.3 °C) over 10 h and maintained for 24 to 288 h. The temperature was controlled within 1 °C of the set value. The pressure in all autoclaves was maintained at 50 MPa by pumping water in or out as needed. When the desired reaction time was reached, the autoclaves were removed from the oven and rapidly cooled to room temperature by quenching in water. The gold tubes were taken out and their contents were analyzed in detail.

### Chemical and isotopic analysis

The gold tubes were cleaned with dichloromethane and then loaded individually into a piercing unit connected to a custom-made glass vacuum line. After evacuation, the vacuum pump was isolated and each gold tube was pierced with a stainless-steel piercing device to release the volatile products into the glass vacuum line. The volatiles in each gold tube were collected in an auxiliary instrument connected to a dual-channel Agilent 7890 N gas chromatograph (GC). The GC oven was held at an initial temperature of 60 °C for 3 min, then ramped from 60 to 190 °C at 25 °C/min, and held at 190 °C for 5 min. Seven columns including HayeSep A 80/100 mesh (0.4 m, 1/8 in OD × 2.1 mm i.d.), HayeSep A 80/100 mesh (5 Ft, 1/8 in OD × 2.1 mm i.d.), Molecular Sieve 5A 60/80 mesh (7 Ft, 1/8 in OD × 2.1 mm i.d.), HayeSep Q 80/100 mesh (3 Ft, 1/8 in OD × 2.1 mm i.d.), Molecular Sieve 5A 60/80 mesh (8 Ft, 1/8 in OD × 2.1 mm i.d.), DB-1 (123–1015(cut) 2 m × 0.32 mm OD × 5um i.d.) and HP-AL/S (25 m × 0.32 mm OD × 8 μm i.d.) were used to separate gases. The carrier gas was helium or nitrogen. The gas content was measured under the temperature of 25℃ and pressure of 1 atm (101,325 pa).

The stable carbon isotope values of the gases were measured using a VG Isochrom II mass spectrometer connected to an Agilent 6890 GC. The column used to separate gases was a Poraplot Q (25 m × 0.32 mm i.d.) and the carrier gas was helium. The heating program for the GC oven included an initial temperature of 50 °C held for 4 min, heating to 190 °C at a rate of 25 °C/min, and holding for 1–5 min. If the replicate values showed a precision of 0.5‰, the average of two measurements was taken.

## Results and discussion

### Chemical and isotopic compositions of gaseous reactants

The chemical compositions of the gaseous reactants in the four gold tubes were similar (Table 1), being mainly composed of CH_4_ (25.49%), C_2_H_6_ (30.01%), C_3_H_8_ (24.87%), and N_2_ (19.64%). The δ^13^C values of the contents of the two gold tubes were measured and found to be similar (Table 1). The average δ^13^C values for CH_4_, C_2_H_6_, and C_3_H_8_ were − 33.30‰, − 26.71‰, and − 28.34‰, respectively.

### Chemical composition of the gaseous products

**Figure 2:**
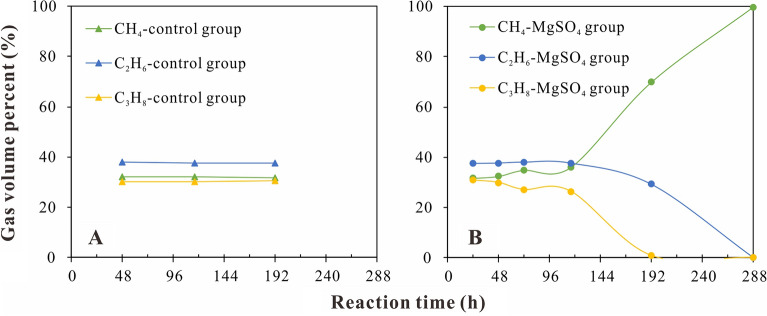
Plots of volume percentages of C_1_–C_3_ alkanes versus reaction time for the control group (**A**) and the MgSO_4_ group (**B**).

Since water could promote the cracking of alkane gases, a control experiment involving water and the alkane gases was conducted to distinguish the effects of the TSR reaction and thermal cracking on the chemical and isotopic composition variations of the gaseous products. In the control experiment, the volume fractions of CH_4_, C_2_H_6_, and C_3_H_8_ showed little variation with increasing reaction time from 48 to 192 h (Fig. 2a), indicating that these alkanes were stable under the thermal simulation conditions at 360 ℃ in the presence of water. However, in the experiment with alkane gases and magnesium sulfate, the gas volume percentages showed great variations when the reaction time exceeded 116 h (Fig. 2b), indicating a destabilizing effect of the MgSO_4_.

**Figure 3:**
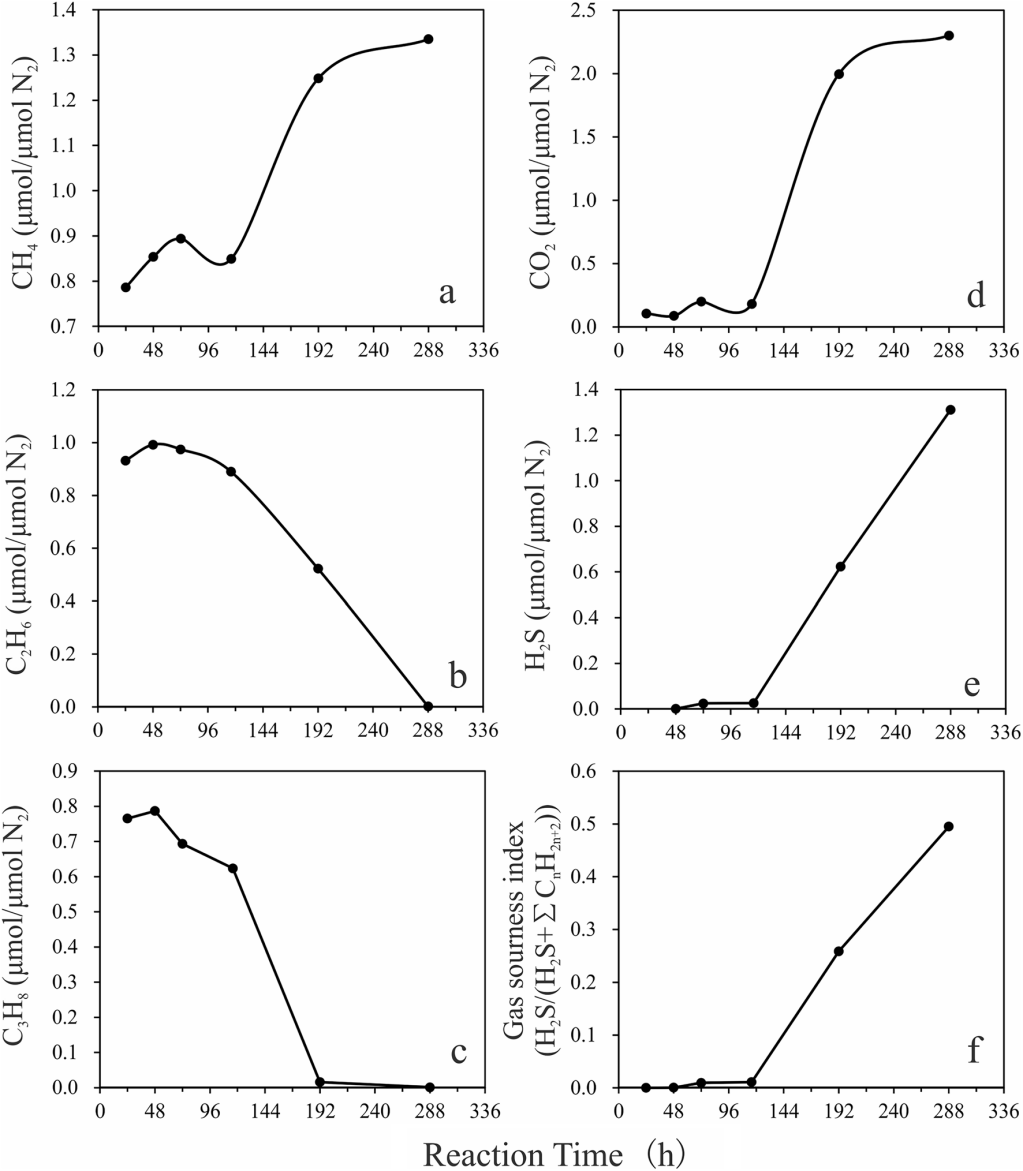
Variations in the contents of CH_4_ (**a**), C_2_H_6_ (**b**), C_3_H_8_ (**c**), CO_2_ (**d**), and H_2_S (**e**) and in the gas sourness index H_2_S/(H_2_S + ΣC_*n*_H_2*n*+2_) (**f**) with reaction time. Contents of gases have been normalized to the amount of N_2_.

To show the variations in the concentrations of the alkane gases and non-hydrocarbon gases (CO_2_ and H_2_S) with increasing reaction times, the gas volume was normalized to the volume of N_2_, as the initial amount of gas introduced into each gold tube varied and N_2_ does not partake in the TSR reaction. As shown in Fig. 3 and Table 2, the contents of the gaseous products showed little variation for reaction time up to 116 h. For the alkane gases, the amount of CH_4_ increased from 0.8 μmol/μmol N_2_ to 0.9 μmol/μmol N_2_, that of C_3_H_8_ decreased from 0.8 μmol/μmol N_2_ to 0.62 μmol/μmol N_2_, and that of C_2_H_6_ decreased from 1.0 μmol/μmol N_2_ to 0.9 μmol/μmol N_2_. For inorganic gases, the amount of CO_2_ increased from 0.1 μmol/μmol N_2_ to 0.2 μmol/μmol N_2_ and the amount of H_2_S increased from 0.001 μmol/μmol N_2_ to 0.026 μmol/μmol N_2_. When the reaction time exceeded 116 h, the gas contents showed large fluctuations. In terms of alkane gases, the content of CH_4_ increased from about 0.9 μmol/μmol N_2_ to 1.33 μmol/μmol N_2_. The main increase (from 0.9 μmol/μmol N_2_ to 1.25 μmol/μmol N_2_) occurred during the period 116–192 h. The content of C_2_H_6_ decreased linearly from 0.9 μmol/μmol N_2_ to 0 μmol/μmol N_2_. The main decrease (from 0.62 μmol/μmol N_2_ to 0.02 μmol/μmol N_2_) in C_3_H_8_ content occurred during the period 116–192 h and a small decrease was observed thereafter. The variation of CO_2_ concentration was similar to that of CH_4_, with the main increase (from 0.2 μmol/μmol N_2_ to 2 μmol/μmol N_2_) occurring during the 116–192 h period. The H_2_S concentration increased linearly from 0.026 μmol/μmol N_2_ to 1.312 μmol/μmol N_2_. The variation of gas sourness index H_2_S/(H_2_S + ΣC_*n*_H_2*n*+2_) was similar to that of H_2_S content, remaining below 0.01 at reaction times up to 116 h and increasing linearly to 0.5 thereafter.

**Table 2:** Chemical composition of gas products in hydrothermal pyrolysis of different experimental conditions.

	Reaction time (h)	Initial loading mass of reactants (mg)		Gas content (μmol)									Gas content (μmol/μmol N_2_)					
		MgSO_4_**·**7H_2_O	H_2_O	CH_4_	C_2_H_6_	C_3_H_8_	C_3_H_6_	N_2_	H_2_	CO_2_	H_2_S	Total gas	CH_4_	C_2_H_6_	C_3_H_8_	CO_2_	H_2_S	Total gas
Control group	48	–	100	104.00	123.22	97.88	-	128.72	1.53	7.74	–	462.70	0.81	0.96	0.76	0.06	–	2.59
	116	–	100	89.25	104.73	83.97	0.05	103.87	0.08	6.94	–	389.42	0.86	1.01	0.81	0.07	–	2.74
	192	–	100	117.38	139.93	114.19	0.07	125.58	0.17	7.73	–	504.70	0.93	1.11	0.91	0.06	–	3.02
MgSO_4_ group	24	600	100	81.74	96.89	79.56	–	103.95	1.68	11.00	–	374.89	0.79	0.93	0.77	0.11	–	2.59
	48	600	100	73.24	85.10	67.51	0.03	85.78	0.03	7.48	0.10	319.35	0.85	0.99	0.79	0.09	0.001	2.72
	72	600	100	69.84	76.15	54.18	0.05	78.13	0.02	15.63	1.92	295.74	0.89	0.97	0.69	0.20	0.025	2.79
	116	600	100	82.66	86.57	60.68	0.04	97.28	0.04	17.67	2.52	347.72	0.85	0.89	0.62	0.18	0.026	2.57
	192	600	100	134.03	56.09	1.72	–	107.33	–	214.28	66.95	580.36	1.25	0.52	0.02	2.00	0.624	4.41
	288	600	100	139.16	0.19	0.13	–	104.25	0.05	239.87	136.74	620.27	1.33	0.00	0.00	2.30	1.312	4.95
Different concentrations of MgSO_4_	72	300	100	95.31	107.58	84.42	0.06	79.95	0.02	5.51	0.28	372.67	1.19	1.35	1.06	0.07	0.004	3.67
	192	50	100	112.50	86.16	12.05	–	97.77	–	46.07	61.70	416.16	1.15	0.88	0.12	0.47	0.631	3.26
	288	50	100	133.04	49.11	0.45	–	91.07	–	86.03	152.59	512.41	1.46	0.54	0.00	0.94	1.675	4.63

### Division of the TSR reaction of natural gas into two stages

**Figure 4:**
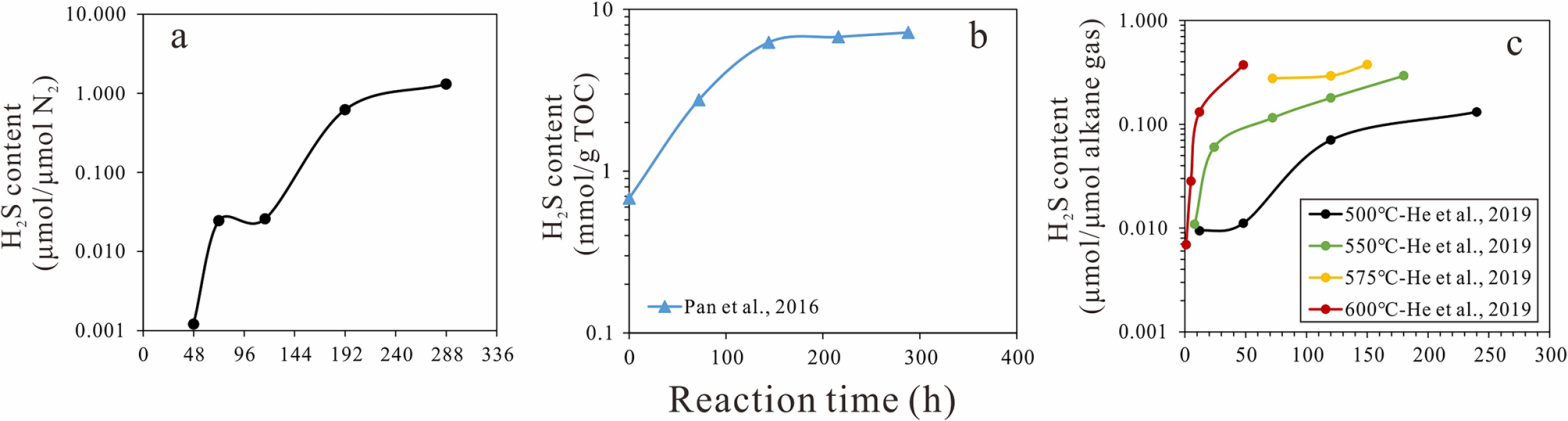
Variations in H_2_S contents with reaction time in this work (**a**), Ref.^25^ (**b**), and Ref.^24^ (**c**).

Through conducting TSR reactions involving C_21_ to C_35_ normal alkanes, it was proposed that the process could be divided into uncatalyzed and catalyzed stages^11^. H_2_S content is the key parameter defining the two stages. During the uncatalyzed stage, the content of H_2_S increases at a slow rate. After reaching a plateau for a certain period, the catalyzed stage starts, during which the H_2_S content increases rapidly. H_2_S catalyzes sulfate reduction through reacting with hydrocarbons to form LSC^10,11,27^. Uncatalyzed stage of TSR reaction is a first-order reaction, and the activation energy for liquid hydrocarbons reacting with HSO_4_^−^ has been determined^11^. Based on the chemical composition of gas produced from the TSR reaction involving natural gases reported by Pan et al.^25^, Xia et al.^26^ found the TSR reaction of gaseous hydrocarbons to be a kinetically controlled non-autocatalytic process. The absence of the required intermediate is believed to be the main cause for the apparent lack of autocatalysis. He et al.^24^ have conducted TSR reactions with gaseous hydrocarbons and investigated the kinetic parameters. They also treated the whole TSR reaction as a non-autocatalytic process. In this study, TSR reactions of gaseous hydrocarbons showed that the production of H_2_S through sulfate reduction with mixed hydrocarbon gas can be divided into two stages (Fig. 4a).

At reaction times up to 116 h, the amount of H_2_S produced by sulfate reduction increases at a slow rate, probably as a result of HSO_4_^−^ ion reduction by hydrocarbons without H_2_S catalysis^11^. Under the experimental conditions, hydrolysis of magnesium sulfate leads to the precipitation of a magnesium hydroxide sulfate hydrate complex and increases the HSO_4_^−^ concentration in solution^26,35^.

1 $$ {\text{5Mg}}^{{{2} + }} + {\text{ 4H}}_{{2}} {\text{O }} + {\text{ 4SO}}_{{4}}^{{{2} - }} = {\text{ Mg}}\left( {{\text{OH}}} \right)_{{2}} \cdot{\text{4MgSO}}_{{4}} \cdot{\text{2H}}_{{2}} {\text{O }} + {\text{ 2H}}^{ + } , $$

2 $$ {\text{H}}^{ + } + {\text{ SO}}_{{4}}^{{{2} - }} = {\text{ HSO}}_{{4}}^{ - } . $$

As the reaction proceeds, H_2_S content generated in a higher rate and accumulates in the system, such that it can eventually catalyze TSR. The variation in H_2_S content with reaction time reported by Pan et al.^25^ was different from that observed here. This might be related to the high H_2_S content in their original gas mixture that was produced by the thermal cracking of kerogen (sulfur content 3.5%) at 450 ℃ (Fig. 4b). The TSR reaction studied by He et al.^24^ covered the whole process. The pyrolysis reaction was conducted under the temperature higher than 500 ℃, leading to high reaction rate (Fig. 4c). And there were few data points in the uncatalyzed stage. Under these conditions, the uncatalyzed stage could be easily neglected.

The reaction mechanism of the catalyzed stage was different from that of the uncatalyzed stage. Compared with the catalyzed stage, the reaction rate in the uncatalyzed stage was significantly slower. Therefore, the kinetic parameters for the two stages should differ greatly. To evaluate the reaction rate and initiation temperature of the TSR reaction involving hydrocarbon gases under geological conditions, further thermal hydro-pyrolysis simulation should be conducted to obtain the kinetic parameters for the uncatalyzed stage.

### Variations in ln(C_1_/C_2_) and ln(C_2_/C_3_)

**Figure 5:**
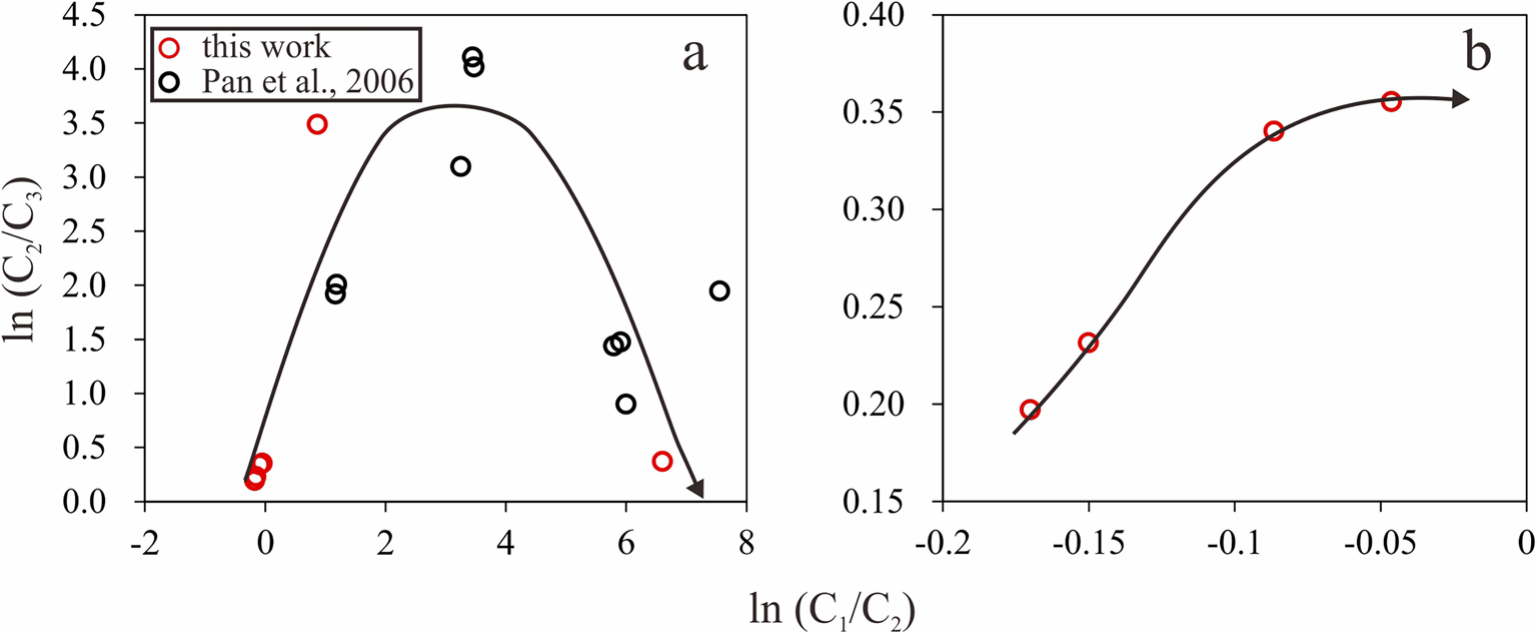
Co-variation of ln(C_1_/C_2_) and ln(C_2_/C_3_) with increasing TSR extent for the whole TSR process (**a**) and the initial uncatalyzed stage (**b**).

A plot of ln(C_1_/C_2_) versus ln(C_2_/C_3_) can be used to distinguish gas origin from the primary cracking of kerogen and secondary cracking of oils^28^. In order to examine the occurrence and extent of the TSR reaction of hydrocarbons and to differentiate TSR-generated gas from thermal cracking gas, a plot of ln(C_1_/C_2_) versus ln(C_2_/C_3_) was used for alkane gases derived from TSR reactions of alkane gases. With increasing extent of TSR reaction, the value of ln(C_1_/C_2_) increased from − 0.17 to 6.61. The value of ln(C_2_/C_3_) initially increased from 0.2 to 3.49 over the period 24–192 h, but then decreased to 0.37 after a reaction time of 288 h (Fig. 5a). Few studies have addressed the TSR reaction of alkane gases and the concentration variations thereof. Pan et al.^25^ conducted the TSR reaction involving mainly alkane gases and small amount of condensate and highly-mature kerogen (prepared by isothermal pyrolysis of kerogen under 450 ℃ for 72 h), variations in ln(C_1_/C_2_) and ln(C_2_/C_3_) with increasing extent of TSR from their work was similar to those reported here (Fig. 5a). For their work, the decrease of ln(C_2_/C_3_) might be related to the TSR reaction of condensate and kerogen. The experiment conducted in this work proved that the TSR reaction involving merely hydrocarbon gases would lead to the decrease of ln(C_2_/C_3_), providing new evidence for identifying gas origin.

During the catalyzed stage of the TSR reaction involving gas, the increase in ln(C_1_/C_2_) is different from that of secondary cracking gas, and the initial increase and subsequent decrease in ln(C_2_/C_3_) are different from those of primary kerogen cracking gas. Both ln(C_1_/C_2_) and ln(C_2_/C_3_) increased with increasing extent of TSR reaction during the uncatalyzed stage (Fig. 5b). This was also different from what was observed for both primary kerogen cracking gas and secondary cracking gas. These results may be taken as evidence for occurrence of the TSR with alkane gases and for interpretation of the origin and migration of gas from sour gas reservoirs.

### Variation of carbon isotope distribution of gaseous products with reaction time

**Figure 6:**
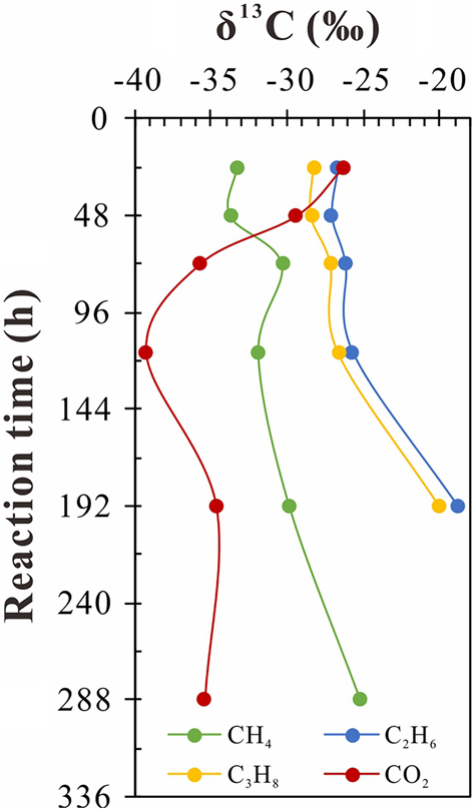
Variations of δ^13^C values of CH_4_, C_2_H_6_, C_3_H_8_, and CO_2_ with reaction time.

Carbon isotopic compositions of methane, ethane, propane, and CO_2_ are shown in Table 3 and Fig. 6. The δ^13^C compositions of the alkane gases became more positive with increasing reaction time (Fig. 6). The δ^13^C values showed small changes at reaction times up to 48 h. During the period 48–116 h, δ^13^C_1_ value increased from − 33.71 to − 31.86‰, δ^13^C_2_ value increased from − 27.12 to − 25.73‰, and δ^13^C_3_ value increased from − 28.42 to − 26.64‰. The δ^13^C value increase was smaller than 2‰. The δ^13^C value increase in the catalyzed stage was significantly greater than that in the uncatalyzed stage, amounting to 6.7‰, 7.0‰, and 6.6‰ for methane, ethane, and propane, respectively. The δ^13^C values for CO_2_ indicated ^13^C depletion with increasing reaction time in the uncatalyzed stage and ^13^C enrichment in the catalyzed stage.

**Table 3:** Carbon isotopic compositions of gas products in hydrothermal pyrolysis of different experimental conditions.

	Reaction time (h)	Initial loading mass of reactants (mg)		δ^13^C (‰, VPDB)			
		MgSO_4_·7H_2_O	H_2_O	CH_4_	CO_2_	C_2_H_6_	C_3_H_8_
Control group	48	–	100	− 33.5	− 36.27	− 27.47	− 28.72
	116	–	100	− 32.59	− 37.3	− 25.73	− 27.58
	192	–	100	− 32.46	− 36.05	− 25.58	− 27.55
MgSO_4_ group	24	600	100	− 33.27	− 26.37	− 26.72	− 28.27
	48	600	100	− 33.71	− 29.44	− 27.12	− 28.42
	72	600	100	− 30.32	− 35.76	− 26.23	− 27.15
	116	600	100	− 31.86	− 39.26	− 25.73	− 26.64
	192	600	100	− 29.83	− 34.68	− 18.86	− 20.05
	288	600	100	− 25.17	− 35.41	–	–
Different concentrations of MgSO_4_	72	300	100	− 34.04	− 38.87	− 28.42	− 29.17
	192	50	100	− 31.23	− 39.14	− 23.64	− 14.07
	288	50	100	− 31.89	− 33.13	− 16.5	–

For natural gases from marine carbonate reservoirs in the northeastern Sichuan Basin, most gases with H_2_S content lower than 0.01 displayed reversed carbon isotope ratios between methane and ethane, whereas most gases with H_2_S contents greater than 0.01 displayed normal isotope ratios between methane and ethane^1^. TSR pyrolysis involving C_9_ hydrocarbon showed reversed carbon isotope ratios between methane and ethane after a reaction time of 24 h at 360 ℃^34^. With increasing extent of TSR, the δ^13^C value of ethane increased and the reversed carbon isotope ratio (δ^13^C_1_ > δ^13^C_2_) changed to a positive sequence (δ^13^C_1_ < δ^13^C_2_)^34^. The reversed carbon isotope δ^13^C_1_ > δ^13^C_2_ was also observed during the uncatalyzed stage of the TSR reaction involving *n*-C_5_ hydrocarbon (under review). However, the reversed carbon isotope ratio was not observed in the uncatalyzed stage of the TSR reaction involving alkane gases in this work. These comparisons suggest that the carbon isotope distribution of alkane gases from carbonate gas reservoirs in the northeastern Sichuan Basin may have been greatly influenced by the TSR reaction of condensate oil.

The observed variation in δ^13^CO_2_ was similar to that reported by Pan et al.^25^. During the uncatalyzed stage (reaction time smaller than 116 h), the decrease in δ^13^CO_2_ with reaction time might be related to the equilibrium isotopic fractionation between gaseous CO_2_ and secondary carbonate precipitation of MgCO_3_^36,37^, to which it is more susceptible in this stage. This is because the amount of CO_2_ generated during the uncatalyzed stage is significantly smaller than that generated during the catalyzed stage. The major positive shift of δ^13^CO_2_ occurred at the catalyzed stage (116–288 h), during which the amounts of ethane and propane decreased sharply (Tables 2,3). The carbon isotopic fractional factor α (*k*^12^/*k*^13^) for alkane gases progressively decreased during the TSR reaction, which would account for the positive shift in δ^13^CO_2_^25^. This mechanism might be partially responsible for the positive shift in δ^13^C for CO_2_ produced in the TSR with natural gases.

### Effects of magnesium sulfate concentration and water content on TSR reaction rate

**Figure 7:**
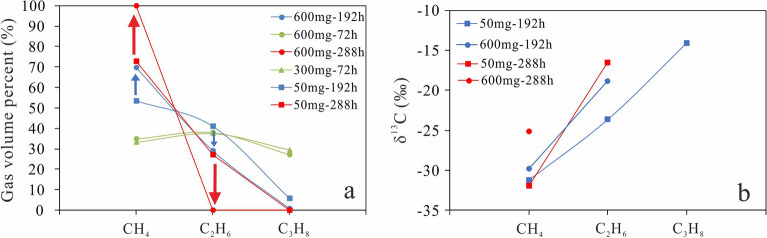
Variation of the volume percentages (**a**) and δ^13^C (**b**) of alkane gases with magnesium sulfate concentration.

With a reaction time of 72 h, the volume percentage of methane was slightly lower and that of propane was slightly higher for a solution containing 300 mg MgSO_4_·7H_2_O, compared with the data for a solution containing 600 mg MgSO_4_·7H_2_O (Fig. 7). The consumption of propane led to the generation of methane during the uncatalyzed stage. The reaction rate in the solution with MgSO_4_·7H_2_O (300 mg) should be lower than that in the solution with MgSO_4_·7H_2_O (600 mg) as the concentration of HSO_4_^−^ and the aqueous volume in the former solution are lower than those in the latter. For the solution with MgSO_4_·7H_2_O (300 mg), the aqueous phase contained 0.24 mmol SO_4_^2−^ and 235 μL water, and the reaction generated 0.48 mmol H^+^. The concentration of HSO_4_^−^ in the solution should be about 1 mol/dm^3^. The concentration of HSO_4_^−^ in the solution with MgSO_4_·7H_2_O (600 mg) was about 1.3 mol/dm^3^ and the water content was about 60% higher at 370 μL. It is expected that the reaction between HSO_4_^−^ and alkane gases take place in the aqueous phase. Therefore, the larger volume of the aqueous phase and higher concentration of HSO_4_^−^ in the solution with MgSO_4_·7H_2_O (600 mg) can be expected to lead to a higher consumption rate of alkane gases.

After a reaction time of 192 h, the volume percentages of methane, ethane, and propane for a solution with MgSO_4_·7H_2_O (50 mg) were 53%, 41%, and 6%, respectively. When the reaction time increased to 288 h, the volume percentage of methane increased to 73% and those of ethane and propane decreased to 27% and about 0%, respectively (Fig. 7a). The volume percentage of methane is significantly lower and those of ethane and propane are higher than those of the alkane gases produced from the solution with MgSO_4_·7H_2_O (600 mg) after the same reaction time (Fig. 7a). The δ^13^C values for methane and ethane were less negative for the solution with MgSO_4_·7H_2_O (600 mg) than for the solution with MgSO_4_·7H_2_O (50 mg) after a reaction time of 192 h (Table 3, Fig. 7b). For the solution with MgSO_4_·7H_2_O (50 mg), the volume of the aqueous phase was about 122 μL and the concentration of HSO_4_^−^ was about 0.33 mol/dm^3^, around a third and a quarter, respectively, of those for the solution with MgSO_4_·7H_2_O (600 mg). The concentration of HSO_4_^−^ and the aqueous volume could greatly influence the reaction rate of the catalyzed stage of TSR involving alkane gases.

It has been reported that when sampling at different sites of Khuff reservoirs in the same gas field at similar depths and temperatures, the extents of TSR reaction involving natural gases was significantly different^21^. Formation waters of petroleum reservoirs have extremely low concentrations of HSO_4_^−^ and the considerable amounts of H_2_S are not likely generated through the oxidation of organic matter by HSO_4_^−^^11^. In nature, it is proposed that [MgSO_4_]_CIP_ is the reactive sulfate specie in petroleum reservoir formation waters^11^. Our experimental results may help to explain that the different extents of TSR of natural gases could be related to different volumes of water and different concentrations of [MgSO_4_]_CIP_. It is estimated that the concentration of [MgSO_4_]_CIP_ in aqueous solutions where TSR occurs ranges from 1.0 × 10^–4^ to 6.5 × 10^–3^ M^11^.The concentration of [MgSO_4_]_CIP_ may vary from place to place due to alteration by the local environment. For example, the salinity (NaCl wt%) varies from about 10 to 25% pre-TSR and from about 4% to 8% post-TSR for carbonate gas reservoirs located in the northeastern Sichuan Basin^38^. Higher salinity would increase the concentration of dissolved sulfate^9^. Some other factors could also affect the concentration of [MgSO_4_]_CIP_, in turn affecting the TSR reaction rate.

## Conclusions

To understand the effect of thermochemical sulfate reduction (TSR) on gaseous hydrocarbons, hydro-pyrolysis involving natural gas and various amounts of MgSO_4_·7H_2_O has been carried out, and the chemical and isotopic compositions of the gaseous products were measured. The following conclusions can be drawn:

1. As the extent of TSR reaction involving wet gas increases, the value of ln(C_2_/C_3_) first increases and then decreases, while that of ln(C_1_/C_2_) increases non-linearly. This is consistent with the results of previous work on TSR reaction involving mainly alkane gases and small amount of condensate and highly-mature kerogen. This work further suggests that the TSR reaction involving merely alkane gases could result in the decrease of ln(C_2_/C_3_). The co-variation of ln(C_1_/C_2_) and ln(C_2_/C_3_) throughout the TSR reaction process can be used to differentiate TSR-influenced gas from primary gas and secondary cracking gas. This is significant for interpreting the origin and migration of gas from sour gas reservoirs.
2. According to the amount of H_2_S generated during the TSR reaction of alkane gases, the process can be divided into uncatalyzed and catalyzed stages. This contradicts a previous hypothesis that the TSR reaction of hydrocarbon gases is a non-autocatalytic reduction process. Future work should determine the kinetic parameters for the uncatalyzed stage to better constrain the occurrence of the TSR reaction of alkane gases.
3. A reversed carbon isotope ratio, δ^13^C_1_ > δ^13^C_2_, was not observed during the uncatalyzed stage of the TSR reaction involving alkane gases, whereas previous studies have indicated δ^13^C_1_ > δ^13^C_2_ in TSR reactions involving C_9_ hydrocarbon. This may suggest that the carbon isotope distribution of gas from carbonate gas reservoirs in the northeastern Sichuan Basin may be affected by TSR reaction of the condensate.
4. Our experimental results have shown that the volume of the aqueous phase and the concentration of magnesium sulfate influence the TSR reaction rate of alkane gases during both the uncatalyzed and catalyzed stages. This could help to explain the previous observation of different TSR reaction extents of hydrocarbon gas recovered from reservoirs at similar depths and temperatures within a single gas field.

## Data Availability

The datasets used and/or analyzed during the current study are available from the corresponding author on reasonable request.
